# New Viral Vector for Superproduction of Epitopes of Vaccine Proteins in Plants

**Published:** 2011

**Authors:** L.G. Tyulkina, E.V. Skurat, O.Yu. Frolova, T.V. Komarova, E.M. Karger, I.G. Atabekov

**Affiliations:** Faculty of Biology, Lomonosov Moscow State University; Belozersky Institute of Physico-Chemical Biology, Lomonosov Moscow State University

**Keywords:** potexviruses, viral vector, foreign epitope, chimeric virus-like particles

## Abstract

The novel viral vectors PVX-CP AltMV and PVXdt-CP AltMV are superexpressors of the capsid protein (CP). These viral vectors were constructed on the basis of the potato virus X (PVX) genome and*Alternanthera*mosaic virus (AltMV) CP gene. The expression, based on the hybrid viral vectors, is genetically safe, since the systemic transport and formation of infective viral particles are blocked. CP AltMV can self-assemble into virus-like particles (VLPs) in the absence of genomic RNA. The vectors can be used for the presentation of foreign peptides (including epitopes of human pathogens) on the surface of the VLP. The N-terminal extracellular domain (M2e) of the influenza virus A M2 protein and its truncated variant (ΔM2e) were used as model heterologous peptides for the construction of the chimeric CP AltMV. Chimeric CP AltMV retains its ability to self-assemble into VLP. The epitopes of the M2 influenza virus protein were not eliminated during the process of accumulation, polymerization and purification of chimeric VLP AltMV, providing evidence of the stability of chimeric VLP with C-terminal heterologous epitopes. It appears that VLP produced by the vectors PVX-CP AltMV and PVXdt-CP AltMV can be used in the field of biotechnology for the presentation of the epitopes of vaccine proteins on their surfaces. The chimeric VLP AltMV with the presented foreign epitopes can be used as candidate vaccines.

## INTRODUCTION

The utilization of plants for the expression and accumulation of foreign (target) proteins (TPs) used in medicine, veterinary science, agriculture, and industry is one of the most promising directions in bioengineering.

Plant cells are superior to those collected from animals or microorganisms in terms of both their technological simplicity and the possibility to simultaneously produce a large amount of inexpensive target products, and proteins produced from plants are completely safe, due to the absence of pathogens that are common to humans and plants. The presence of post-translational modification systems in plants typically ensures correct conformation formation of target proteins through disulfide bonds and glycosylation.

One of the most efficient methods for the rapid production of significant amounts of target proteins in the cytoplasm of infected cells is the use of autonomously replicating recombinant viral vectors. The high rate of viral genome replication allows to achieve high copy numbers of transcripts from foreign genes in the infected cell’s cytoplasm. Therefore, the average efficiency of a viral expression system is higher than the efficiency upon stable plant transformation or the transient expression using nonviral vectors by two orders of magnitude [1, 2]. Viral vectors constructed on the basis of plant RNA viruses (tobamo-, potex-, como-, bromo-, and potyviruses) are the most frequently used. [3].

There are two major strategies for producing target proteins using a viral vector:

1) The TP gene is expressed under the control of the viral promoter, yielding an individual protein. This can be based on the “gene insertion strategy,” when a TP gene is placed under the control of the duplicated subgenomic promoter (e.g., the coat protein gene [2, 4–6]) or on the “gene substitution strategy,” when a viral gene (most frequently the coat protein gene and/or the genes responsible for viral transport) is substituted for the TP [7–11]. This approach facilitates the accumulation of the target protein at quantities amounting to at least 10% of the total amount of soluble plant proteins, over a short period of time. Vectors based on the phytoviral genome have been successfully applied in the production of proteins for medical use (including vaccine proteins) in plants. This strategy was used to synthesize the hepatitis B virus core protein (HBcAg) and calicivirus capsid protein, which are capable of forming stable virus-like particles stimulating the humoral and cellular immune responses [12, 13];

2) The strategy of “fusion” of the nucleotide sequence of the TP gene, or its fragment, and the viral gene or its fragment. Here, the viral gene of the capsid protein and the TP gene are typically expressed as a single translational frame. However, there are limits to the size of the inserted foreign sequence. Extensive polypeptides are usually linked to the principal protein via a flexible bridge, via proteolysis sites, via the sequence of 2A peptide from the foot-and-mouth disease virus, etc. [14–17].

Studies of the molecular mechanisms of the activation of the adaptive immune response indicate that peptides, rather than entire proteins, are more likely to be responsible for the activation of T- and B-lymphocytes [18]. Both synthetic and recombinant peptides are unstable and possess weak antigenic activity [19]; however, binding to high-molecular weight supports with a high immunogenicity results in the enhancement of the immunogenicity and stability of these peptides [18].

An example of such supports are the capsid proteins (CP) of plant viruses, which are capable of forming viral and/or virus-like nanoparticles which can be used for the presentation of pathogen epitopes on their surface. These nanoparticles have a stable and regularly repeating structure that facilitates the induction of strong cellular and humoral immune responses [20–23].

The strategy was used to construct viral vectors that produce viruses with chimeric CP in plants. The epitopes of antigens of the *Staphylococcus aureus* , foot-and-mouth disease virus, hepatitis C virus, papillomavirus, human immunodeficiency virus, influenza virus [24–33], and many other viruses (see reviews [3, 34]) were presented on the surface of these virions. 

However, viral vectors based on full-length genomes (capable of systemic transport and formation of infective viral particles) are considered unsafe for use in bioengineering. When producing the target proteins, it is impossible to eliminate the probability of penetration of recombinant viruses into the environment, followed by uncontrolled propagation of the genetic material. 

The capsid proteins of certain animal and plant viruses retain their ability to form stable capsids, virus-like particles (VLPs), in the absence of a viral genome. The use of VLPs for efficient presentation of epitopes foreign to the immune system [21, 35–37] on their surface can resolve the problem of bio-safety as relates to vaccine protein production. Today, special attention is paid to the construction of vector systems that express capsid proteins forming VLPs free of any RNA impurities. Only such VLPs are believed permissible for application in bio- and nano-engineering [38, 39].

This study focuses on the construction of a system of genetically safe viral vectors based on potex virus genomes for the production of VLPs that present pathogen epitopes on their surface in plants.

## EXPERIMENTAL

**Media, reagents, enzymes, and synthetic oligonucleotides**

 In this study, we used *Escherichia coli* XL-1 Blue (Stratagene, USA) and *Agrobacterium tumefaciens* GV3101 from the collection of the Department of Virology, Moscow State University. The recombinant DNAs were cloned in *E. coli * XL-1 Blue cells through the conventional procedures [40] using restriction endonucleases, DNA ligase, and Taq and Pfu polymerases (Fermentas, Lithuania and SibEnzyme-M, Russia). The oligonucleotides were synthesized by Sintol (Russia). The following synthetic oligonucleotides were used for cloning: CP AltMV-XhoI-p (CTAGCTCGAGATGTCCACTCCATTTCCTCAA), CP AltMV-XbaI-m (CGTCTAGATTACTCCGGTGGTGGGAGGTATTGA), PVX-R-Avr2-p (TGCACAGATTTTCCTAGGCAC), PVX-R-XhoI-m (AGCTCTCGAGCTTATTCAAATCTCTAAGGTA), PVX-3ntr-XbaI-p (AGCTTCTAGACTACGTCTACATAACCGACGC), Oligo(dT) _24_ -Kpn-m (AGCTGGTACCTTTTTTTTTTTTTTTTTTTTTTTT), PVX-Kpn-(dT) _12_ -3ntr-m (AGCTGGTACCTTTTTTTTTTTTATATTATTCATACAATC), PVX-Xba-cpxho-3ntr-p (AGTCTCTAGTCGAGGCGTTCAGGAACA), CP AltMV-evetpirn-XbaI-m (CGTCTAGATTAGTTTCTGATGGTGTTTCACCCTCCGGTGGTGGGAGGTA), CP AltMV-M2E-m (TTTCCACCTCTGTCAAGAGTGACTCCGGTGGTGGGAGGTA), M2E-XbaI-m (CGTCTAGATTAGTCGGATGAGTCGTTGCATCT), M2E-p (TCACTCTTGACAGAGGTGGAAACACCAATCAGAAACGAGTGGG), and M2E-m (GTCGGATGAGTCGTTGCATCTGCATCCCCACTCGTTTCTGATT). The validity of the obtained constructs was verified by automatic sequencing plasmid DNA samples at GenoTekhnologiya (Russia). 

**PVX-CP AltMV and PVXdt-CP AltMV vector constructs**

 Binary hybrid vectors were constructed in several stages using intermediate constructs (ICs). 

**IC 1**

 – The fragment from the plasmid PVX-201 containing the 35S promoter, PVX replicon with the duplicated subgenomic promoter, but without the transcription terminator of the nopaline synthase gene (Tnos-terminator) at the HindIII/EcoRI sites, was transferred into the pBIN 19 binary vector. 

**IC 2**

 – The AltMV capsid protein gene was obtained by PCR on a matrix of cDNA-copy of the 3’-terminal region of the AltMV genomic RNA using primer pair CP AltMV-XhoI-p and CP AltMV-XbaI-m and sub-cloned into the Cambia 6963 vector at the XhoI/XbaI restriction sites.

**IC 3 **

– For convenient cloning, the XbaI restriction site flanking the subgenomic promoter of 25 kDa protein PVX was substituted for the XhoI restriction site. For this purpose, the cDNA fragment of PVX encoding the C-terminal region of the viral polymerase was obtained by PCR on the PVX-201 matrix using the PVX-R-Avr2-p and PVX-R-XhoI-m primer pair. The synthesized fragment was cloned at the Avr2/XhoI sites into the pGEM3-11369(polio)x2 vector containing the subgenomic promoter of the 25 kDa protein gene; the sequence encoding the C-thermal region of PVX polymerase and the CP U1 tobacco mosaic virus (TMV) with a duplicated poliovirus epitope. The procedure was used to delete the sequence of the U1 TMV CP gene from the pGEM3-11369(polio)x2 vector and design the XhoI restriction site at the 3’-terminus of the subgenomic promoter of the 25 kDa protein.

**IC 4**

 – Three variants of 3’-NTR were obtained by PCR on the PVX-201 matrix using three primer pairs: PVX-3ntr-XbaI-p and Oligo(dT) _24_ -Kpn-m were used to synthesize 3’-NTR of PVX (A) _24_ ; primers PVX-3ntr-XbaI-p and PVX-Kpn-(dT) _12_ -3ntr-m, to synthesize 3’-NTR of PVX (A) _12_ ; and primers PVX-Xba-cpxho-3ntr-p and PVX-Kpn-(dT) _12_ -3ntr-m, to synthesize 3’-NTR of PVX p/cp(A) _12_ . 

Following cleavage by XbaI/Kpn restrictases, the synthesized DNA fragments corresponding to different variants of 3’-NTR were cloned into the pBlueScript II SK ^+^ plasmid at XhoI–(XbaI)–KpnI sites, simultaneously with the CP AltMV gene that had been removed at a preliminary stage from IC 2 at the XhoI/XbaI sites. 

**IC 5**

 – The DNA fragment corresponding to CP AltMV with the adjacent 3’-NTR was removed from IC 4 at the XhoI/KpnI restriction sites and cloned into IC 3 at the XhoI–(KpnI)–SacI sites, simultaneously with the DNA fragment that corresponded to the Tnos-terminator and had been removed at a preliminary stage at the KpnI/SacI sites from the pGEM subclone containing the Tnos-terminator.

At the last stage of cloning, the DNA fragment from IC 5 at the XhoI/SacI or AvrI/SacI site was transferred into the IC 1 that had undergone prior treatment with SalI/SacI or AvrI/SacI restrictase.

**Obtainment of vector constructs expressing chimeric capsid proteins of AltMV**

** IC 6 **

– The CP AltMV gene containing the sequence encoding the ΔM2e-variant was obtained by PCR on the matrix of a cDNA copy of the 3’-terminal region of AltMV genomic RNA using the CP AltMV-XhoI-p and CP AltMV-evetpirn-XbaI-m primer pair, and it was subcloned into the Cambia 6963 vector at the XhoI/XbaI restriction sites. The CP AltMV gene encoding the full-length М2е domain was obtained by PCR via three stages, using the pair of synthetic oligonucleotides М2Е-p and М2Е-m, and two primer pairs (CP AltMV-XhoI-p and CP AltMV-М2Е-m, CP AltMV-XhoI-p and M2E-XbaI-m) and subloned into the Cambia 6963 vector at the XhoI/XbaI restriction sites. 

**IC 7**

 – In IC 5, the sequence of the viral capsid protein was substituted at the XhoI/XbaI sites for the sequences of chimeric capsid proteins from IC 6.

At the final cloning stage, the DNA fragments from IC 7 at site XhoI/SacI or AvrI/SacI were transferred to the IC 1 that had been preliminarily treated with the restrictase SalI/SacI or AvrI/SacI.

**Agroinjection**

 Agrobacteria *A. tumefaciens* (strain GV3101) were transformed by recombinant plasmids using the freeze–thawing procedure [41]. The agrobacteria containing recombinant binary vectors were cultivated overnight on a incubator shaker at 28 ^0^ С in an LB medium containing 50 mg/l of rifampicin, 50 mg/l of kanamycin, and 25 mg/l of gentamicin. The cells were deposited by centrifugation at 4000 g for 5 min and then resuspended in an agroinjection buffer containing 10 mM of Mes (pH 5.5) and 10 mM of MgSO _4_ . *Nicotiana benthamiana* leaves were injected with an agrobacterial suspension ( *OD*_600_  = 0.2) using a needle-free syringe. Following the agroinjection, the plants were grown under a daylight lamp subjected to 16 h of light per day at a temperature of 22 ^0^ С. In order to suppress posttranscriptional gene silencing, the agroinjection of plants was performed in the presence of the p19 suppressor gene of the tomato bushy stunt virus. 

**Analysis of expression of the AltMV capsid protein upon agroinjection**

 The preparations from agroinjected *N. benthamiana* leaves were homogenized in three-five volumes of the extraction buffer (10 mM Tris, pH 8.0, containing 5 mM EDTA). The obtained suspension was clarified by centrifugation at 12000 g for 15 min. An equal volume of denaturating buffer for subsequent application onto polyacrylamide gel was added to the supernatant. The buffer for applying the samples on SDS-PAGE gel contained 60% of glycerol, 20% of β-mercaptoethanol, 10% of sodium dodecyl sulfate, 250 mM Tris-HCl buffer, pH 6.8, and 1% bromophenol blue. The analyzed samples were heated at 95 ^0^ С for 15 min and fractionated by electrophoresis in 12% SDS-PAGE gel using the Laemmli procedure [42], followed by Coomassie R-250 staining. The chimeric capsid viral proteins synthesized in *N. benthamiana * leaves were identified via Western blot analysis as previously described [43], using polyclonal antibodies to CP AltMV and/or M2e-epitope and secondary antibodies conjugated to horseradish peroxidase (Sigma). The reaction products were visualized via chemiluminescence, using the ECL system (Amersham Biosciences). 

**Extraction of AltMV capsid proteins from plant tissue**

**Fig. 1:**
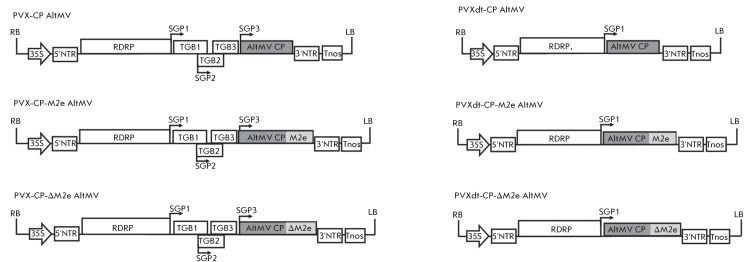
Schematic representation of hybrid viral vectors constructed on the basis of the potato virus X (PVX) genome and *Alternanthera* mosaic virus (AltMV) CP gene. RDRP – gene of viral RNA-dependent RNA polymerase; TGB1, TGB2, TGB3 – triple gene block; sgp – sub-genomic promoter are indicated by arrows; CP AltMV– gene of *Alternanthera* mosaic virus capsid protein; М2e – N-terminal extracellular domain of the M2 protein of the influenza virus; ΔM2e - truncated variant of the М2e; 35S - promoter for the 35S RNA of CaMV; Tnos - terminator of nopaline synthase; NTR - non-translatable region; LB – left border and RB – right border of the T-DNA. The initial polygenomic vector PVX-CP AltMV and the truncated replicon vector PVXdt-CP AltMV contained DNA fragments encoding full-length native CP AltMV. The complete genome chimeric vectors (PVX-CP-M2e AltMV and PVX-CP-ΔM2e AltMV) and chimeric replicon vectors (PVXdt-CP-M2e AltMV, PVXdt-CP-ΔM2e AltMV) encoded chimeric coat proteins: CP-M2e AltMV and CP-ΔM2e AltMV. The above constructs were cloned into the binary vector and used to infect plants via agrobacteria.

 On the sixth - eighth day after agroinjection, the leaf material was ground in the extraction buffer (10 mM Tris, pH 8.0, 5 mM EDTA) in order to achieve a homogeneous suspension. The resulting mixture was centrifuged at 12000 g for 15 min, followed by the collection of the supernatant containing capsid proteins. For the polymerization of CP AltMV and formation of VLPs, a 0.5 M citrate buffer, pH 4.0, was added to the supernatant until a concentration of 25 mM was achieved, followed by incubation for 40 min at room temperature. The pseudovirions from the plant extract were deposited by ultracentrifugation at 100 000 g for 120 min, or using polyethylene glycol (8% PEG 6000, 2% NaCl, 25 mM citrate buffer, pH 4.0). The precipitates were suspended in a 25 mM citrate buffer (pH 4.0), incubated for 40 min for the correcting polymerization, and then clarified by centrifugation at 12000 g for 15 min. The resulting preparations of chimeric VLPs were subjected to an enzyme immune assay and electron microscopy analysis. 

**Electron microscopy**

 The samples prepared by the conventional negative contrast procedure using a 1% uranyl acetate solution were viewed on a JEM-1011 transmission electron microscope (JEOL, Japan). The images were made using a Gatan Erlangshen ES500W digital camera and Gatan Digital Micrograph software × 250000 *.*

## RESULTS AND DISCUSSION

**Hybrid viral vectors**

**Fig. 2:**
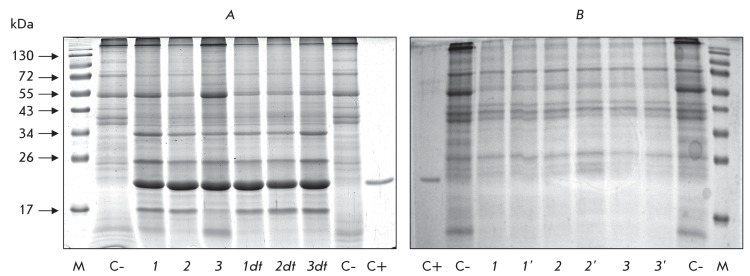
AltMV capsid protein production in *N. benthamiana* leaves agroinjected with viral vectors PVX-CP AltMV and PVXdt-CP AltMV with differing 3’-NTR (1,1dt, 3’-NTR PVX (А) _24_ ; 2,2dt, 3’-NTR PVX p/cp (A) _12_ ; 3,3dt, 3’-NTR PVX (А) _12_ ). Coomassie stained 12% SDS-PAGE gel loaded with 2.5 mg of total protein extracted from agroinjected leaves; C+, CP AltMV, 0.5 µg; C-, non-inoculated leaf (negative control); M, protein molecular weight markers. (A) – agroinjected leaves, 8 dpa; (B) – upper systemic leaves, 16 dpa (1, 2, 3) and 20 dpa (1’, 2’, 3’) after agroinjection of lower leaves

 Potato virus X and the *Alternanthera* mosaic virus belong to the genus *Potexvirus * (potexviruses), family Flexiviridae. The particles of typical PVX are flexible filamentous virions of helical structure with a length of 515 nm and a diameter of 13.5 nm. Approximately 1,300 identical capsid protein subunits form the polar PVX helix with a pitch of 3.6 nm. The viral RNA is located between the helix turns; each turn comprises 8–9 CP subunits. The particles have a hollow central axial channel with a diameter of 3 nm [44, 45]. An expression system based on potexvirus genomes was designed using the popular vector PVX-201 containing a complete cDNA copy of the PVX UK3 genome cloned between the 35S promoter of the cauliflower mosaic virus (CaMV) and the Tnos-terminator [4]. AltMV serologically close, but not identical, to the papaya mosaic virus (PMV) was used as a capsid protein donor [46]. CP PMV differs from the CP of the typical representative of potexviruses (PVX) in terms of the former’s ability to form virus-like particles *in vitro* with helical symmetry, without the participation of RNA [47]. The data of the electron microscopy analysis of CP AltMV preparations indicate that the AltMV capsid protein is also capable of *in vitro * formation of VLPs. 

Two types of hybrid viral vectors, PVX-CP AltMV (complete genome variant) and PVXdt-CP AltMV (minireplicon variant), were constructed on the basis of the PVX and AltMV genomes. The PVX-CP AltMV genome is controlled by the 35S promoter and Tnos *-* terminator and contains 5’- and 3’-untranslated regions of PVX RNA, the gene of RNA-dependent RNA polymerase of PVX, and the triple block of PVX transport genes. The CP AltMV gene is expressed under the control of the subgenomic promoter of CP PVX. The PVXdt-CP AltMV is also controlled by the 35S promoter and Tnos-terminator and contains 5’- and 3’-untranslated PVX RNA regions, and the gene of RNA-dependent RNA polymerase of PVX. However, it does not contain the triple gene block, like in the case of the construct described in [10]. In viral vectors PVXdt-CP AltMV, the gene of the AltMV capsid protein is controlled by the subgenomic promoter of the 25 kDa PVX protein. 

**Fig. 3:**
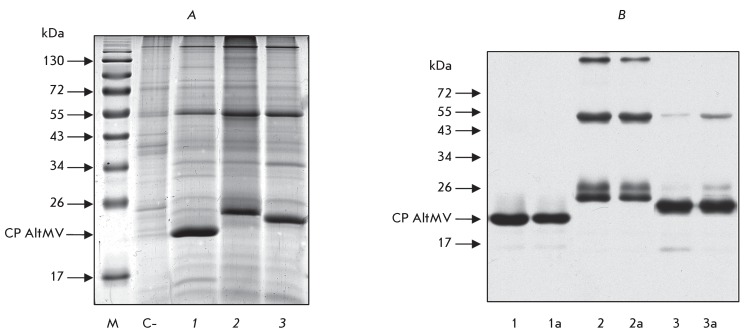
Production of CP AltMV and chimeric AltMV capsid protein in *N. benthamiana* leaves agroinjected with viral vectors: PVX-CP AltMV (1) and PVXdt-CP AltMV (1a); PVX-CP-M2e AltMV (2) and PVXdt-CP-M2e AltMV (2a),  PVX-CP-ΔM2e AltMV (3) and PVXdt-CP-ΔM2e AltMV (3a); 6 dpa. A – Coomassie stained 12% SDS-PAGE gel loaded with 1.5 mg of total protein extracted from agro-injected leaves. C-, no agro-injected leaf, negative control; B – Western blot analysis of CP AltMV and chimeric AltMV capsid protein production in agroinjected leaves using polyclonal antibodies to CP AltMV. M, protein molecular weight markers. The positions of CP AltMV are indicated by arrows.

**Fig. 4:**
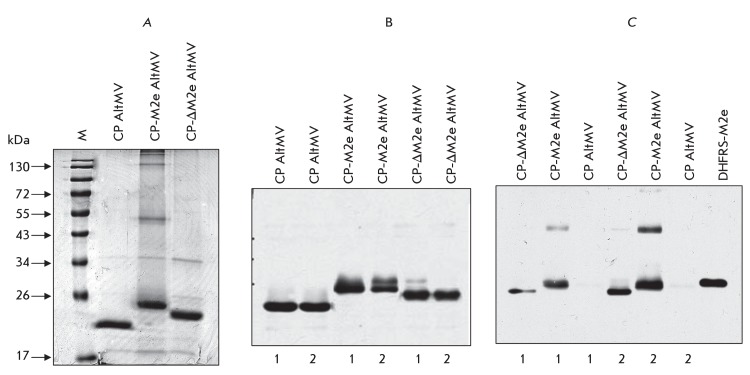
Analysis of VLP preparations formed by CP AltMV and chimeric AltMV CP with M2 influenza virus protein epitopes (M2e and ΔM2e) obtained in *N. benthamiana* leaves and purified by PEG precipitation (1) or by ultracentrifugation (2). (A) – Laemmli electrophoresis of VLP preparations purified by ultracentrifugation. Coomassie stained 12% SDS-PAGE gel. (B) – Western blot analysis of VLP preparations using polyclonal antibodies to CP AltMV. (C) – Western blot analysis of VLP preparations using polyclonal antibodies to influenza A virus M2e-epitope. Recombinant protein DHFRS-M2e was used as a control for the M2e-epitope.

It is known that, for efficient synthesis of the target protein, the viral vector needs to contain a certain set of *cis* -acting elements in the 3’-untranslated region, which determine the following: the affinity to replicase, to facilitate transcription and translation, and to provide a maximum expression level of foreign genes. Since the 3’-terminal CP PVX gene in hybrid viral vectors is substituted by the CP AltMV gene, three model variants of 3’-NTR were used to construct the vectors: 3’-NTR of PVX (A) _24_ ; 3’-NTR of PVX (A) _12_ ; and 3’-NTR of PVX p/cp (A) _12_ . The 3’-NTR of PVX (A) _12_ corresponds to the 3’-terminal region of the full-length infectious cDNA copy of the PVX genome (PVX-201). The 3’-NTR of PVX (A) _24 _ does not contain an ATAAAT sequence; however, the poly(A) tract is increased from 12 to 24 A. The 3’-NTR of PVX p/cp (A) _12_ contains the corresponding PVX-201 poly(A) tract; however, the 3’-NTR is increased by 60 nucleotides at its 5’-terminus, due to the CP PVX gene that is adjacent to the 3’-NTR. 

**Fig. 5:**
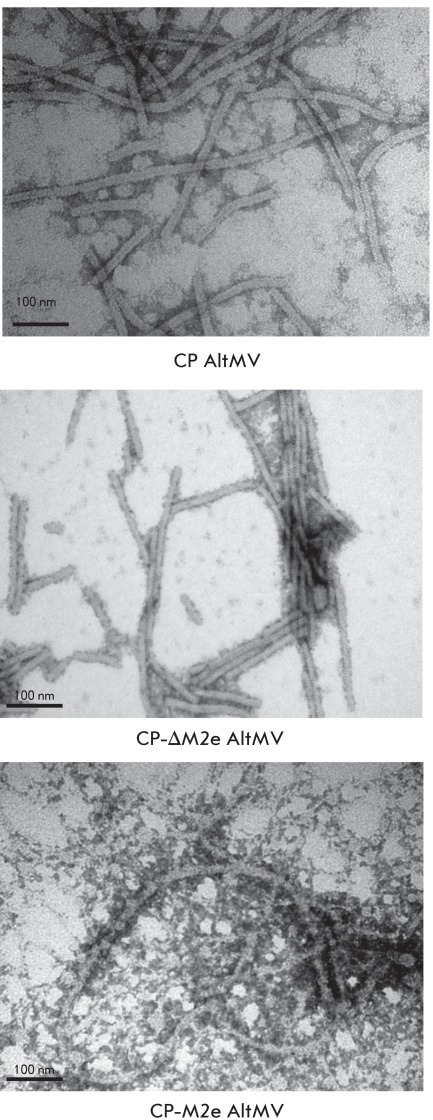
Electron micrographs of AltMV VLP and chimeric AltMV VLP preparations obtained in *N. benthamiana* leaves. Polymers of CP AltMV were negatively stained with a 1% uranyl acetate solution. The bar is 100 nm. × 250000.

Thus, two types of hybrid viral vectors ( *Fig. 1* ) were constructed on the basis of the PVX genome at the first stage: PVX-CP AltMV and mini-vector-replicon PVXdt-CP AltMV, with three variants of 3’-NTR for each vector. All constructs were cloned into the binary pBIN19 vector for further infection of *N. benthamiana * plants with agrobacteria. 

**Replication of hybrid viral vectors in **

*N. benthamiana* **leaves**

The efficiency of the expression of the CP AltMV gene using various vectors was determined via the accumulation of the capsid protein in *N. benthamiana* leaves on the sixth - eighth day after agroinjection. It should be noted that, in all the constructs under discussion, the replication of the hybrid viral vector PVX-CP AltMV in *N. benthamiana * plants led to the accumulation of CP AltMV in approximately identical amounts as were determined for CP PVX upon mechanical inoculation of the virus to plants (over 1 mg per 1 g of green material). AltMV is typically accumulated in plants in lower concentrations (approximately 340 µg per 1 g of green material). 

No distinctions were detected in the efficiency of CP production when using different 3’-NTR. The deletion of the 60 nucleotide 3’-terminal fragment from the CP PVX gene did not result in a reduction in the CP AltMV level, contrary to the data presented in [10]. It can reasonably be assumed that the existing homology between the 3’-terminal regions of the CP PVX and CP AltMV genes is sufficient to provide an efficient performance of PVX polymerase. Similar results, namely, the absence of preference for any 3’-NTR variant and a similar level of accumulation of the recombinant CP AltMV, were also obtained upon replication of PVXdt-CP AltMV viral vectors ( *Fig. 2A* ). Vector constructs with only a single 3’-NTR variant (3’-NTR of PVX (A) _12_ ) were subsequently used ( *Fig. 1* ). 

The mini-vectors PVXdt-CP AltMV were not superior to the PVX-CP AltMV vectors in terms of accumulation of the recombinant AltMV capsid protein in *N. benthamiana * leaves on days 6–8 following the agroinoculation in the presence of the gene-silencing suppressor (the p19 protein gene of the tomato bushy stunt virus) ( *Fig. 2A).*

It is known that intercellular and systemic transport of PVX in plants is regulated by four genes, including the triple gene block (TGB) and CP [4]. No recombinant CP AltMV was detected in the systemic, non-agroinjected *N. benthamiana * leaves that were agroinoculated with hybrid viral PVX-CP AltMV vectors on days 16–20 ( *Fig. 2B* ). Thus, the substitution of the CP PVX gene in recombinant hybrid viral vectors for the CP AltMV gene results in disruption of the systemic transport of the hybrid virus. 

It was demonstrated via electron microscopy analysis of the extracts from *N. benthamiana * leaves agroinoculated with the hybrid viral vectors PVX-CP AltMV and PVXdt-CP AltMV that the recombinant capsid protein of AltMV can form extensive virus-like particles (see below), which can be used to present foreign epitopes on their surface. A UV absorption spectrum within 225–339 nm of the VLP-CP AltMV preparation isolated from the plant extract attests to the absence of RNA in it (the data are not presented). 

When used for presentation of pathogen epitopes, it is noteworthy that VLPs with helical symmetry present a certain advantage over VLPs with icosahedral symmetry; specifically, they possess a greater number of subunits per VLP. Therefore, it is possible that they can present a larger number of pathogen epitopes by chimeric CP in VLP.

**Construction of hybrid viral vectors expressing chimeric capsid proteins of AltMV**

 The N-terminal domain of the influenza A virus М2 (М2е) protein and its truncated variant (ΔM2e), which are responsible for the triggering of the protective immune response, were used as a model foreign peptide for construction of the chimeric CP AltMV [48]. The influenza A virus matrix (M2) protein is considered a promising candidate for the design of an antiviral vaccine, since the amino acid sequence of the ectodomain of this protein (M2e) is highly conserved and has remained almost unaltered since the human influenza A virus was first isolated in 1933 [49]. A consensus synthetic amino acid sequence of the M2 protein ectodomain was proposed on the basis of a computer analysis of 55 isolates of the human influenza A virus [50]. 

Since the conformational structure of CP AltMV is still as yet unknown, the search for the optimal insertion site (an insertion site which ensures the presentation of a heterologous epitope on the capsid protein surface whilst having a minimal effect on CP conformation without impeding the formation of polymeric structures) was carried out using the DNAStar software package. Finally, the C-terminal localization of the M2e-epitope and the ΔM2e variant within the chimeric CP AltMV were selected. The nucleotide sequence of the M2e-epitope and the ΔM2e variant was constructed on the basis of the corresponding consensus amino acid sequence of the N-terminal domain of the influenza A virus M2 protein, using the synonymous codons that occur most frequently in the genome of the PVX and AltMV capsid proteins.

The chimeric CP AltMV genes encoding the full-length M2 protein ectodomain (23 amino acid residues, M2e-epitope), SLLTEVETPIRNEWGCRCNDSSD, and the truncated variant, ΔМ2е (8 amino acid residues, EVETPIRN) fused to the C-terminus of CP (CP-M2e AltMV and CP-ΔМ2е AltMV), were obtained by PCR. Cloning sequences of chimeric capsid proteins in the hybrid viral vectors PVX-CP AltMV and PVXdt-CP AltMV were performed to replace the sequences of the viral capsid proteins.

Thus, four hybrid viral vectors containing the genes of the chimeric capsid proteins of AltMV ( *Fig. 1* ) were constructed on the basis of the PVX genome: PVX-CP-M2e AltMV and its mini-variant PVXdt-CP-M2e AltMV; PVX-CP-ΔМ2e AltMV and its mini-variant PVXdt-CP-ΔМ2e AltMV. These constructs were used to transform competent cells of agrobacteria to infect *N. benthamiana * plants *.*

**Expression of chimeric capsid proteins of AltMV in **

*N. benthamiana* **leaves**

Hybrid viral vectors based on the PVX genome which encode the genes of the chimeric capsid proteins of AltMV (CP-М2е and CP-ΔМ2e) were agroinjected into *N. benthamiana * leaves. Six to eight days following the agroinjection, the synthesis of chimeric proteins was quantitatively assessed via fractionation of the soluble proteins in SDS-PAGE gel and Coomassie staining ( *Fig. 3A* ). The chimeric capsid viral proteins were identified via a Western blot analysis using polyclonal antibodies to CP AltMV or М2е-epitope ( *Fig. 3B* ). Same as for CP AltMV, no differences in the accumulation of chimeric capsid proteins were observed upon replication of the full-length hybrid viral vector or its mini-variant. The production of CP-M2e and CP-ΔМ2e depended upon the individual features of the plant, the layer a leaf belonged to, and seasonal conditions. Nevertheless, it follows from the electrophoregrams shown in *Fig. 3* that the level of accumulation of the chimeric capsid proteins of AltMV (CP-М2е and CP-ΔМ2e) in plant leaves is comparable to the level of recombinant CP AltMV and is equal to over 1 mg (in some experiments, up to 3 mg) per 1 g of green material. 

The chimeric capsid proteins of AltMV with M2e-epitope and the ΔМ2e-variant, similar to the initial capsid protein of AltMV, form extensive virus-like particles with pH decreasing to 4.0–4.9 under conditions of low ionic strength of a solution. A chimeric VLP preparation can be isolated from the plant extract by ultracentrifugation or precipitation with polyethylene glycol. The results of the enzyme immune assay and electrophoretic and electron microscopy analyses of chimeric VLP preparations are shown in *Figs. 4, 5* . As follows from the presented data, epitopes of the influenza A virus M2 protein are not eliminated during the accumulation, polymerization, and purification, which attests to the stability of the chimeric CP AltMV when the foreign epitope localizes at the C-terminus. The fact that the foreign epitope does not impede the polymerization of the chimeric CP AltMV upon C-terminal localization indicates the conformational differences between the C-terminal regions CP AltMV and CP PVX [51]. 

## CONCLUSIONS

Our study aimed to design a system that could be used to present heterologous epitopes (pathogene epitopes) on the surface of the virus-like particles formed by the capsid protein of the phytovirus. The hybrid viral vectors PVX-CP AltMV and PVXdt-CP AltMV, with a number of advantages over the initial PVX and AltMV viruses, were constructed on the basis of the PVX genome and the CP AltMV gene.

1) The level of accumulation of the recombinant capsid protein of AltMV upon agroinjection of *N. benthamiana * leaves with hybrid viral vectors is as high as 1 mg/g of plant material, which considerably exceeds the accumulation upon mechanical inoculation of a natural host; 

2) The substitution of the CP PVX gene in hybrid viral vectors for the CP AltMV gene results in the suppression of systemic transport of the hybrid virus and disruption of viral particle formation;

3) The ability to form VLPs is ensured by the feature of the AltMV capsid protein; CP PVX is incapable of forming VLPs.

The results presented point to the potential application of the viral vectors PVX-CP AltMV and PVXdt-CP AltMV in bioengineering in order to produce vaccine proteins in plants. The AltMV capsid protein that is accumulated at a high concentration and forms virus-like particles in the absence of genomic RNA can be used for efficient presentation of epitopes of human and animal infectious agents on the VLP surface. Chimeric capsid proteins of AltMV with model heterologous peptides, epitopes of the influenza A virus M2 protein, are also capable of forming stable VLPs. The expression system based on the hybrid viral vectors PVX-CP AltMV and PVXdt-CP AltMV is genetically safe. The use of this vector system allows one to avoid both the spontaneous vertical and horizontal transmission of plant infection and the unregulated spread of genetic material into the environment. 

## Abbreviations

PVX: potato virus X
AltMV: *Alternanthera*mosaic virus
CP: capsid protein
dt: deletion of triple gene block
PVX-CP AltMV: hybrid viral vector based on PVX genome and CP AltMV
M2e: ectodomain of influenza A virus M2 protein
ΔM2e: truncated variant of M2e
CP-M2e AltMV, CP-ΔM2e AltMV: chimeric CP AltMV with epitope of M2 protein
VLP: virus-like particles
PCR: polymerase chain reaction
NTR: Non-translated region
